# Depression, anxiety, insomnia and interleukins in the early postpartum period

**DOI:** 10.3389/fpsyt.2023.1266390

**Published:** 2023-09-28

**Authors:** Ewa Drozdowicz-Jastrzębska, Anna Mach, Michał Skalski, Piotr Januszko, Zoulikha Jabiry-Zieniewicz, Marcin Siwek, Zbigniew Maciej Wawrzyniak, Maria Radziwoń-Zaleska

**Affiliations:** ^1^Department of Psychiatry, Medical University of Warsaw, Warsaw, Poland; ^2^1st Department of Obstetrics and Gynecology, Medical University of Warsaw, Warsaw, Poland; ^3^Department of Affective Disorders, Jagiellonian University Medical College, Cracow, Poland; ^4^Faculty of Electronics and Information Technology, Warsaw University of Technology, Warsaw, Poland

**Keywords:** depression, anxiety, insomnia, interleukins, postpartum period

## Abstract

**Background:**

Some new mothers have been shown to suffer from anxiety and depression associated with insomnia during the postpartum period. Our study assessed the impact of demographic, psychopathological, and biochemical factors on the incidence of depression in women during the early postpartum period.

**Methods:**

A total of 119 women were evaluated at 24–48 h postpartum with the following psychometric scales: Hamilton Depression Rating Scale (HDRS), Edinburgh Postnatal Depression Scale (EPDS), Hamilton Anxiety Rating Scale (HARS) and Athens Insomnia Scale (AIS). In addition, blood was drawn to assay interleukin 6 (IL-6) and interleukin 10 (IL-10).

**Results:**

The factors that had the greatest impact on the risk of postpartum depression detected with the HDRS were high HARS scores and evidence of insomnia in the AIS. There were no significant differences in IL-6 or IL-10 levels in women with and without depression (based on either HDRS or EPDS scores) and insomnia (based on AIS) after childbirth. Considering demographic factors, divorced and single women were shown to be at higher risk of postpartum depression (based on EPDS scores).

**Limitations:**

Small sample size and short observation span.

**Conclusion:**

This study highlights the relationship between postpartum depression and both anxiety and insomnia and emphasises the importance to assess symptoms of anxiety and sleep quality as part of screening in women at risk of postpartum depression.

## 1. Introduction

The birth of a baby is usually a joyous event both for the mother and her family. Nonetheless, childbirth itself is associated with a number of psychological and physical challenges. The most common mental disturbance is postpartum depression, affecting 7–19% women who give birth (1–3). The International Classification of Diseases (ICD-10) defines a birth-related depressive episode as one that occurs within 6 weeks after childbirth (4). According to The American Psychiatric Association, a postpartum-onset major depressive disorder (MDD) is one whose symptoms manifest within 4 weeks after delivery (5). However, elevated risk of depression has been reported to persist for the whole year after childbirth (6).

Postpartum depression may adversely affect the course of puerperium and impair mother–infant bonding (7–9). There are ongoing research efforts to identify potential biomarkers of depression, i.e., compounds that could help diagnose depression in women after childbirth on the molecular level and help predict the risk of new-onset, recurrent, and potentially treatment-refractory depression (10). Candidates for such biomarkers include certain hormones (estradiol, progesterone, cortisol) and inflammatory proteins (11, 12).

Although the clinical presentation of postpartum depression does not differ considerably from depression at other periods of life, some studies indicate high rates of bipolar traits and a high prevalence of anxiety and insomnia in the former, which produce substantial clinical complications (13–15).

### 1.1. Sleep disturbances considered alongside postpartum depression and anxiety

Sleep disturbances that occur in women after childbirth adversely affect the well-being of both the mothers and their babies. Problems with sleep often accompany depression and anxiety (4, 5, 16, 17). Disturbed sleep may be a prodromal sign of new-onset or recurrent depressive episodes (18–20).

Poor sleep quality has been linked to postpartum symptoms of depression and anxiety and with postpartum depression without anxiety (21, 22). A study conducted by Okun found that women who had more severe symptoms of anxiety and depression scored higher on the PSQI (Pittsburgh Sleep Quality Index), taking into account the influence of variables such such as depression and anxiety disorders during pregnancy (23).

Sleep disturbances seem to be a risk factor for mental health deterioration in women during the peripartum period. Therefore, sleep quality assessment should be a part of screening in women at a high risk of developing postpartum depression and anxiety disorders after childbirth.

### 1.2. Depression considered alongside postpartum anxiety disorders

Epidemiological data indicate the prevalence of anxiety disorders during the postpartum period to be approximately 28% (24, 25). The factors that have been linked to increased peripartum anxiety levels are higher levels of education, history of depression, pre-term delivery, negative experiences associated with delivery and the first week after delivery, an inconsolable baby, poor coping mechanisms, inadequate support from the partner, and poor health (26). An anxiety disorder in the mother has been reported to increase the risk of obstetric complications, the stress of being a parent, and behavioral problems in the child (27).

Since there are no separate diagnostic criteria for anxiety disorders during the postpartum period, the symptoms are similar to those associated with anxiety disorders at other periods of life and include concerns about being a mother, about the baby’s safety, excessive apprehension for the baby’s health, and fear of hurting the baby. In moderation, such concerns may be beneficial; however, when they are excessive, they lose their adaptive function and may hinder the baby’s care (28).

### 1.3. Interleukins and depression

Classic models of depression show the role of increased hypothalamic–pituitary–adrenal (HPA) axis activation; however, many authors additionally emphasize the importance of the immune system in the etiology of depression, particularly the inflammatory response system (IRS) (29, 30). IRS activation affects the HPA, which alters the metabolism of 5-hydroxytryptamine (5-HT) and catecholamines, and may increase the risk of depression in animals and humans (31).

Recent studies demonstrated increased levels of many pro-inflammatory cytokines, including interleukins (IL), such as IL-6, in individuals suffering from depression (32, 33). Moreover, individuals with depression were shown to have abnormal IL-10 levels, resulting in an upset pro- and anti-inflammatory cytokine balance (32, 34).

One study conducted in 1,034 adults showed that individuals with MDD have significantly elevated IL-6 levels (*p* ≤ 0.001) and IL-10 levels (*p* ≤ 0.001) in comparison with those in the control group (35). A meta-analysis of studies on the differences in cytokine and chemokine levels in individuals with depression demonstrated elevated levels of many inflammatory markers, including IL-6 and IL-10 (36). Patients with depression were also found to have lower IL-10 levels and an increased IL-6/IL-10 ratio in comparison with healthy individuals, which suggests disturbances in the regulation of cytokine secretion in depression (37). These studies indicate that IL-6 and IL-10 might be potential markers of depression.

There are few studies evaluating the relationship between postpartum depression and cytokine levels. One of such studies demonstrated increased IL-6 levels in women with depression during late pregnancy and early postpartum period (30), whereas another study showed a decreased interferon-gamma/IL-10 ratio, which may indicate impaired cell-mediated immunity (38).

Pregnancy alters the level of immune system activation in order to ensure maternal tolerance of the fetus. Consequently, there may be a relationship between immune parameters and postpartum mood disorders. Boufidou et al. demonstrated a relationship between the levels of IL-6 in the cerebrospinal fluid and symptoms of depression within the first 4 days after childbirth (*p* = 0.035) (39). Other authors reported elevated serum IL-6 levels in patients with anhedonia and a major depressive episode (40).

There is evidence that serum tryptophan availability in women during late stages of pregnancy and first days after childbirth is significantly lower than that in non-pregnant women. The final stage of pregnancy and the early postpartum period are associated with immune system activation. Low levels of kynurenine towards the end of pregnancy result from low plasma tryptophan levels. Inflammation may increase tryptophan degradation along the kynurenine pathway, which results in an increased kynurenine/tryptophan ratio at the end of pregnancy and during the early postpartum period. Consequently, symptoms of depression and anxiety during the early postpartum period may be due to increased tryptophan catabolism to kynurenine, which may result from immune system activation (41).

### 1.4. Interleukins and anxiety disorders

Research on anxiety disorders and pro-inflammatory cytokines is scarce, with conflicting results. The association between anxiety and inflammation has been demonstrated in a recent study that showed elevated C-reactive protein (CRP) levels in suicidal patients with anxiety disorders (42). An analysis of 36 biomarkers (including leptin, brain-derived neurotrophic factor [BDNF], and tryptophan) demonstrated higher rates of anxiety disorders in individuals with metabolic disturbances (43).

The relationship between anxiety and markers of micro-inflammation may vary, depending on age and sex (44). One study revealed an inverse relationship between CRP and fibrinogen levels and symptoms of anxiety in healthy women. However, no such relationship was observed in men.

Animal studies showed an increase in IL-6 levels with the associated anxiety in response to stress (45) and a relationship between elevated IL-6 levels during pregnancy and increased risk of anxiety behaviors in the offspring (46).

### 1.5. Interleukins and sleep

Sleep and the circadian rhythm regulate the functioning of the immune system and general well-being. Prolonged periods of short sleep and the associated stress response induce constant production of non-specific pro-inflammatory cytokines and contribute to reduced immunity (47).

Many studies suggest that IL-6 is a “sleep factor,” with the circadian pattern of IL-6 release shown to correlate with periods of sleep and sleepiness. According to Vgontzas, IL-6 seems to be a mediator of sleepiness, and its circadian rhythm reflects the homeostatic drive for sleep (48).

In a study by Irwin et al. sleep disturbance and long sleep duration have been shown to be associated with increased CRP and IL-6 levels. There was no association between either sleep deprivation or short sleep duration and either CRP, IL-6, or TNFα levels (49). Another study showed that a 14-day period of fragmented sleep increased the risk of convulsions in rats and modulated the production of IL-1β and IL-6 in the brain (50). A stydy by Wright et al. showed that chronic circadian misalignment was associated with significant increases in TNF-α, IL-10, and CRP levels (*p* < 0.05) (51).

The potential effects of poor sleep quality during pregnancy on inflammation and the associated risk of obstetric complications are still poorly understood. The few studies on this subject assessed sleep disturbances and inflammatory markers in pregnant women. Okun reported poor sleep quality to be associated with elevated IL-6 levels (52–54) and symptoms of insomnia and poor sleep quality to be associated with dysregulation of several cytokines (53). These findings were consistent with those reported by Blair et al. (55). Okun suggested that sleep disturbances during the early stages of pregnancy lead to obstetric complications by exacerbating inflammation – which may inhibit trophoblast invasion and impair the associated remodeling of placenta-supplying maternal blood vessels (56).

One of the few studies on sleep disturbances after childbirth demonstrated that sleep duration of ≤5 h per day after childbirth was associated with higher IL-6 levels than those in individuals sleeping >5 h per day. Short sleep duration during the first year postpartum was associated with elevated IL-6 levels at 3 years postpartum (57).

## 2. Materials and methods

The purpose of this study was to assess the effect of demographic, psychopathological, and biochemical factors on the prevalence of symptoms of depression and anxiety in postpartum women.

A total of 119 women who gave birth at the First Faculty and Department of Obstetrics and Gynecology of the Medical University of Warsaw in the period 2013–2016 were included in the study. The exclusion criteria were age under 18 years and unstable physical condition. All patients had given their consent to participate in this study. All patients were examined; this included structured history-taking, demographic data, and psychometric scales. The data on previous depressive episodes and insomnia during pregnancy were obtained during a structured history-taking interview. All psychometric examinations were conducted by the same specialist in psychiatry, who is the first author of this article.

*Symptoms of depression* were assessed with the Hamilton Depression Rating Scale (HDRS24), with a score of ≥10 points adopted as the cut-off score for depression, and the Edinburgh Postnatal Depression Scale (EPDS), with the score of ≥12 points adopted as the cut-off.

*Symptoms of anxiety* were assessed with the Hamilton Anxiety Rating Scale (HARS), with each item scored from 0 (not present) to 4 (severe) points and the total score ranging from 0 to 56 points, with the score of <18 points indicating mild, 18–24 mild-to-moderate, and 25–30 points moderate-to-severe symptoms of anxiety.

*Sleep disturbances* were assessed with the Athens Insomnia Scale (AIS), with the score of ≥8 points adopted as the cut-off for insomnia, the value validated for the Polish population by Fornal-Pawłowska et al. 2011 (Fornal-Pawłowska) and a general sleep disturbance questionnaire (Ogólny Kwestionariusz Zaburzeń Snu) – a standard questionnaire for assessing sleep disturbances developed at the Sleep Disturbance Outpatient Clinic of the Nowowiejski Hospital.

*Interleukin levels were assessed in venous blood* collected into S-Monovette tubes (SARSTEDT, REF 01.1604.001) with heparin as the coagulant. The centrifuged citrated plasma was stored at ≤ − 20°C until the time of testing.

Citrated plasma samples were analyzed quantitatively for cytokines IL-6 and IL-10 with an R&D SYSTEMS reagent kit (catalog number IL-6 HS600B; IL-10 HS100C; BDNF DBDOO) using an enzyme-linked immunosorbent assay (*ELISA*). The minimum detectable doses were 0.039 pg./mL for IL-6, 0.09 pg./mL for IL-10, and 20 pg./mL for human free BDNF. The blood tests were conducted according to the manufacturer’s protocol. Optical density was read with microplate readers Multiscan GO (Thermo Scientific).

All tests were conducted in patients 24–48 h after childbirth.

### 2.1. Statistical analysis

The statistical analysis of quantitative variables was conducted with the use of descriptive statistics, such as means, standard deviations, percentiles, and correlations. The Shapiro–Wilk test was used to test whether the distribution of the analyzed quantitative variables deviated from a normal distribution. Since the distribution of the evaluated quantitative variables was shown not to be normal, groups of samples were compared with an unpaired two-sample Wilcoxon test (WilcoxonRank_Sum test). Relationships between categorical variables were evaluated with the use of contingency tables and the chi-square test or Fisher’s exact test for small sample sizes.

The logistic regression generalized linear model (GLM) was employed in multivariate analysis. Odds ratio was used as effect size. The optimal model was selected based on the Akaike information criterion (AIC) statistic.

Values of *p* of less than 0.05 were considered statistically significant.

Statistical analysis calculations were conducted with the use of SAS v.15.2.

This study was approved by the Ethics Committee at the Warsaw Medical Academy (a former name of the Medical University of Warsaw) (approval No. KB/12/2012).

## 3. Results

### 3.1. Univariate analysis

A total of 119 women, aged between 20 and 42 years (mean age 31.01 ± 4.2), were included in the study.

HDRS scores assessed 24–48 h postpartum demonstrated:

Depression in 14.3% of women (*n* = 17, mean age 31 ± 3.95, median 31).

No symptoms of depression in 85.7% of women (*n* = 102, mean age 31 ± 4.2, median 31).

EPDS scores assessed 24–48 h postpartum demonstrated:

Depression in 11.8% of women (*n* = 14, mean age 32.36 ± 2.73, median 32).

No symptoms of depression in 88.2% of women (*n* = 105, mean age 30.84 ± 4.39, median31).

Seventy-one women (59.6%) had a vaginal delivery, and 48 women (40.34%) had a Cesarean section. Breastfeeding was reported by 89.6% of women; 91.6% of the deliveries were full-term, and 8.4% of the deliveries were pre-term.

No relationship was observed between depression and the following variables: age, education, gravidity, parity, mode of delivery (Cesarean section vs. vaginal delivery), breastfeeding, pre-term delivery.

Unemployed women had higher rates of postpartum depression based on HDRS than employed women (36% vs. 12%); however, this difference did not reach statistical significance (*p* = 0.0536).

Among those women who met the EPDS depression criteria, divorced/separated women were more prone to depression (*p* = 0.0303). Detailed demographic characteristics of the study population have been presented in Table 1.

**Table 1:** Demographic characteristics.

	*N*	Women with depression (HDRS ≥10)	Value of *p*	Women with depression (EPDS ≥12)	Value of *p*
*Marital status*					
single	23	5 (22%)	NS	2 (9%)	**0.0303**
married	88	11 (13%)		9 (10%)	
divorced	6	1 (17%)		3 (50%)	
*Household conditions*					
living with family/other members	116	17 (15%)	NS	14 (12%)	NS
alone	1	0 (0%)		0 (0%)	
*Education*					
primary/vocational	4	1 (25%)	NS	0 (05%)	NS
secondary	15	1 (7%)		1 (7%)	
higher	98	15 (15%)		13 (13%)	
*Source of income*					
employed	106	13 (12%)	0.0536	13 (12%)	NS
unemployed	11	4 (36%)		1 (9%)	

The bold values represent statistically significant.

### 3.2. Psychometric scale results

The mean HARS score in the study group was 3.5 ± 3.69, (median 3.00, minimum 0, maximum 18). Only one patient scored ≥18 points, which was the lower threshold of mild symptoms of anxiety.

Forty-one patients (34.5%) achieved AIS scores of ≥8. The AIS-based criteria for insomnia were met by a significantly greater proportion of women diagnosed with depression based on the HDRS (10 out of 17; 58% of women) than women without HDRS score-based postpartum depression (31 out of 102; 30.3% of women) (*p* = 0.0224). There were no differences in the rates of insomnia among women diagnosed with postpartum depression based on the EPDS.

Women diagnosed with depression based on their HDRS scores achieved significantly higher scores than women without depression both in the HARS (9.5 points vs. 2.5 points, *p* < 0.0001) and in the AIS (9.1 vs. 5.7 points, *p* = 0.0096). Women diagnosed with depression based on the EPDS scored significantly higher in the HARS (9.2 points vs. 2.8 points, *p* < 0.0001) than women without depression. Those patients also scored higher in the AIS; however, the difference was not statistically significant (8.8 vs. 5.9 points, *p* = 0.0572) (Table 2).

**Table 2:** Anxiety and insomnia scores in women who developed (1) and who did not develop (0) postpartum depression (HDRS and EPDS).

Parameter	Scale	*N*	Mean	SD	Median	Minimum	Maximum	Value of *p*
	HDRS≥10							
HARS	0	102	2.5	2.4	2	0	11	**<0.0001**
	1	17	9.5	4.4	10	1	18	
AIS	0	102	5.8	3.9	5	0	19	**0.0096**
	1	17	9.1	5.1	9	0	17	
	EPDS≥12							
HARS	0	105	2.8	2.7	2	0	12	**<0.0001**
	1	14	9.2	5.2	10.5	1	18	
AIS	0	105	5.9	3.9	5	0	19	0.0572
	1	14	8.8	5.7	7.5	0	17	

The bold values represent statistically significant.

Data on the previous history of depression were obtained for 110 patients. Ten percent had a history of a depressive episode. Out of the patients with a history of depression, 36% met the HDRS score-based criterion for postpartum depression. Out of the patients with a negative depression history, 13% developed HDRS score-based postpartum depression. Those patients who had a self-reported history of depression were also more likely to develop depression based on HDRS scores; however, the differences between these groups were not statistically significant (*p* = 0.0656).

Out of the patients with a positive history of depression, 27% met the EPDS score-based criterion of depression. Out of the patients with a negative history of depression, 11% met the EPDS score-based criterion for depression. The difference between these subgroups was not statistically significant.

Data on previous history of insomnia were obtained for 100 patients. According to these data, 36% patients had a history of insomnia during pregnancy, with 56% of those also meeting the adopted criterion for postpartum insomnia. Out of 64% women who reported no insomnia during pregnancy, 27% met the criterion for insomnia after childbirth. The patients who reported insomnia during pregnancy were significantly more likely to meet the AIS score-based criterion for insomnia during the postpartum period (*p* = 0.0052).

Out of the patients who had a history of depression, 54% met the AIS score-based criterion of insomnia postpartum. Out of the patients who had no history of depression, 33% developed AIS score-based insomnia after childbirth. The differences between these subgroups were not statistically significant.

Women who developed AIS score-based postpartum insomnia were more likely to score higher in the HARS – this difference was statistically significant (4.39 ± 3.62 vs. 3.06 ± 3.66, *p* = 0.0118).

Blood tests for IL-6 and IL-10 were conducted in 104 and 86 patients, respectively, within 24–48 h after delivery.

The median IL-6 level was 14.05 pg./mL (min. 4.8, max. 53.7) in women with HDRS score-based depression in comparison with 12.75 pg./mL (min.1.67, max. 98.4) in women without depression. The median IL-10 level was 0.99 pg./mL in women with depression (min. 0.79, max. 3.24) and 1.02 pg./mL (min. 0.78, max. 19.4) in women without depression. The difference between IL-6 and IL-10 levels in women with and without HDRS score-based depression was not statistically significant.

The median IL-6 level was 14.7 pg./mL (min. 4.8, max. 53.7) in women with EPDS score-based depression in comparison with 11.8 pg./mL (min. 1.67, max. 98.4) in women without depression. The median IL-10 levels were 1.07 pg./mL (min. 0.79, max. 3.24) and 1.01 pg./mL (min. 0.78, max. 19.4) in women with and without depression, respectively. The difference between IL-6 and IL-10 levels in women with and without EPDS score-based depression was not statistically significant.

The median IL-6 levels in women with and without AIS score-based insomnia were 10.65 pg./mL and 14.95 pg./mL, respectively, and the median IL-10 levels in those two groups were 0.99 pg./mL and 1.04 pg./mL, respectively. The differences between IL-6 and IL-10 levels in women with and without insomnia were not significant.

There was no correlation between IL-6 or IL-10 levels and HARS scores.

### 3.3. Multivariate analysis

Multivariate analysis helped identify the parameters that affected the rates of HDRS and EPDS score-based depression.

#### 3.3.1. Hamilton Depression Rating Scale

In the presented model, the following variables affected the risk of developing HDRS score-based depression: anxiety measured with the HARS and insomnia measured with the AIS (Table 3).

**Table 3:** Multivariate model; odds ratios for variables affecting the rates of HDRS score-based depression.

Effect	Odds ratio	95% LCL	95% UCL	value of *p*
HARS [v + 1 vs. v]	1.65	1.34	2.04	<0.0001
AIS [>7 vs. 1–7]	3.52	0.82	15.08	0.0898

AIS, Athens Insomnia Scale; HARS, Hamilton Anxiety Rating Scale; LCL, lower confidence limit; UCL, upper confidence limit; v, value.

**Figure 1:**
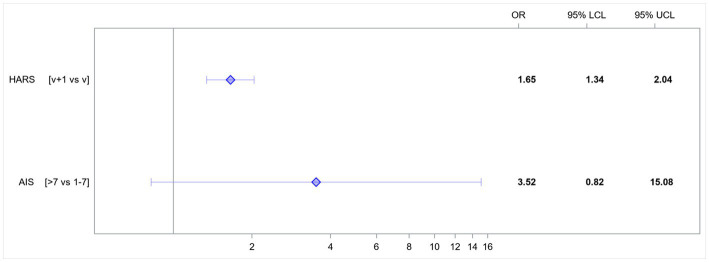
Odds ratios (OR) for variables affecting the rates of HDRS score-based depression. AIS, Athens Insomnia Scale; HARS, Hamilton Anxiety Rating Scale; LCL, lower confidence limit; UCL, upper confidence limit; v, value.

An increase in the HARS score by 1 point increased the risk of developing HDRS score-based depression 1.65-fold. AIS score-based insomnia increased the risk of developing HDRS score-based depression 3.5-fold (Figure 1).

#### 3.3.2. Edinburgh Postnatal Depression Scale

In the presented model, the following variables affected the risk of developing EPDS score-based depression: HARS score-based anxiety and the patients’ marital status (Table 4).

**Table 4:** Multivariate model; odds ratios for variables affecting the rates of EPDS score-based depression.

Effect	Odds ratio	95% LCL	95% UCL	value of *p*
HARS [v + 1 vs. v]	1.47	1.23	1.75	<0.0001
Marital status [single vs. married]	0.59	0.09	3.79	0.0974
Marital status [divorced vs. married]	11.83	1.13	123.32	0.0270

HARS, Hamilton Anxiety Rating Scale; LCL, lower confidence limit; UCL, upper confidence limit; v, value.

**Figure 2:**
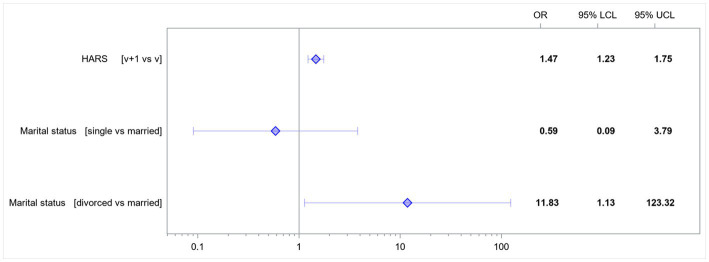
Odds ratios for the variables affecting the rates of EPDS score-based depression. HARS, Hamilton Anxiety Rating Scale; LCL, lower confidence limit; UCL, upper confidence limit; v, value.

An increase in the HARS score by 1 point increased the risk of developing EPDS score-based depression 1.47-fold. Single women had a 0.59-fold lower risk of developing EPDS score-based depression than married women. Divorced women had a 11.83-fold higher risk of developing EPDS score-based depression than married women (Figure 2).

## 4. Discussion

This study showed depression in 14.3% of women evaluated with the HDRS and in 11.8% of women evaluated with the EPDS. These proportions of depression are consistent with those reported by other authors, which range from 10 to 20% of women after childbirth, depending on the adopted criteria (1, 3). Studies in the Polish population showed postpartum depression in 15.2% of patients (15).

Our study showed that the patients diagnosed with insomnia after childbirth also achieved higher HARS scores.

HPA hyperactivity is a well-known neuroendocrine abnormality in depression (58), potentially playing a major role in the pathogenesis of anxiety disorders and sleep dysregulation (58, 59). Many studies indicate a common pathogenic pathway for depression and anxiety disorders. There have been also reports on the common etiology and pathogenesis of depression and sleep disturbances (60); however, there are few studies in women after childbirth.

Our study demonstrated a relationship between postpartum depression and symptoms of anxiety and insomnia. The factors that were found to have the greatest impact on the risk of developing postpartum depression in the HDRS, were higher HARS scores and AIS score-based insomnia. These conclusions are consistent with those reported by other authors, who emphasize the fact that symptoms of anxiety and insomnia are often part of the presentation of postpartum depression. Women with symptoms of anxiety alone or insomnia alone may be at a higher risk for postpartum depression and thus should be closely monitored for symptoms of depression in the weeks following delivery.

The strongest risk factor for postpartum depression is a positive history of depression before and during pregnancy (61). Our study showed that patients with a history of depression were more likely to develop HDRS and EPDS score-based depression, though the differences between the subgroups were not significant.

Other important risk factors for postpartum depression include prenatal anxiety disorders, stressful life events, low self-esteem, poor social support, a low income, and inadequate support from the partner (62). The factor that increased the risk of depression in women who scored ≥12 points in the EPDS was marital status – with divorced and separated women at a considerably higher risk of developing postpartum depression. These findings are consistent with those by other authors, who report that inadequate support from the partner increases the risk of postpartum depression (61, 63).

Considering the reported relationship between inflammation and oxidative stress in affective disorders (64), we had expected higher levels of the pro-inflammatory cytokine IL-6 and lower levels of the anti-inflammatory cytokine IL-10 after childbirth in women who met the depression criteria; however, our study showed no significant differences in the levels of these interleukins between patients with and without depression.

Earlier studies demonstrated plasma IL-6 levels to be elevated in chronic stress, which may contribute to neurobehavioral complications, including anxiety disorders, and affect the brain phenotype in the offspring (45). Ramirez studied the relationship between inflammation in the mother and predisposition to anxiety disorders in the offspring. The study was conducted on Japanese macaques and showed that high levels of IL-6 in the third trimester of pregnancy were associated with lower amygdala volume in the offspring at the age of 4 months and a more rapid increase in amygdala volume between the age of 4 and 36 months. The offspring of those females whose IL-6 levels were elevated during pregnancy showed more anxiety-like behaviors at the age of 11 months (46). Consequently, IL-6 seems to affect brain development; however, the mechanism remains unknown.

Our study showed no relationship between IL-6 levels and an increased severity of anxiety symptoms. These results are consistent with those of another study, which demonstrated that individuals with generalized anxiety disorder (GAD) had lower IL-6 levels; moreover, no relationship was found between GAD and other markers of inflammation (65). One of the studies by Wisner demonstrated that two-thirds of patients who met the EPDS score-based criteria of a postpartum depressive episode also exhibited anxiety disorders, most commonly GAD (66). The discrepant results on the association of IL-6 and symptoms of anxiety may be explained by the fact that individual types of anxiety disorders are associated with different bio-behavioral mechanisms and not all of them are associated with chronic low-grade inflammation. Nazzari has posited that inflammation plays a role in the pathogenesis of depression, but not in that of anxiety, in women after childbirth (67).

In our study, 36% of patients reported insomnia. This result is consistent with that of a study by Wołyńczyk-Gmaj conducted in the Polish population, where 39% of patients were diagnosed with insomnia (68).

The patients who had insomnia during pregnancy were more likely to report insomnia after childbirth. Recent years saw an increased number of studies on sleep disturbances during and after pregnancy. The results of our study are consistent with those of the few earlier studies, which showed that insomnia during pregnancy increases the risk of insomnia in the postpartum period and may also increase the risk of postpartum depression (11, 69, 70).

## 5. Conclusion

1. The variables that had the greatest effect on the risk of developing HDRS score-based postpartum depression were higher HARS scores and AIS score-based insomnia.
2. The variables that had the greatest effect on the risk of developing EPDS score-based postpartum depression were higher HARS scores and patients’ marital status.
3. Women who met the HDRS score-based criterion for depression were also more likely to meet the insomnia criterion (*p* = 0.0224).
4. Women who had a history of depression were more likely to meet the HDRS and EPDS score-based criteria of depression; however, the differences between these subgroups were not significant.
5. There were no significant differences in IL-6 or IL-10 levels in women with and without depression after childbirth (based on either HDRS or EPDS scores).
6. There were no significant differences in IL-6 or IL-10 levels in women with and without AIS score-based insomnia after childbirth.
7. There was no relationship between IL-6 or IL-10 levels and HARS scores.
8. The patients with insomnia during pregnancy were significantly more likely to meet the AIS score-based criterion of insomnia after childbirth (*p* = 0.0052).
9. Women with AIS score-based insomnia after delivery scored higher in the HARS (*p* = 0.0118).
10. There was no relationship between postpartum depression and such variables as age, education, source of income, gravidity, parity, mode of delivery, breastfeeding, pre-term delivery.
11. A study with a larger sample size is needed to confirm these findings.
12. An additional point of clinical assessment will be performed in the continuation of the study at 3–7 days postpartum.

## Data availability statement

The raw data supporting the conclusions of this article will be made available by the authors, without undue reservation.

## Ethics Statement

The studies involving humans were approved by the Ethics Committee at the Warsaw Medical Academy (a former name of the Medical University of Warsaw) (approval No. KB/12/2012). The studies were conducted in accordance with the local legislation and institutional requirements. The participants provided their written informed consent to participate in this study.

## Author contributions

ED-J: Investigation, Resources, Methodology, Writing – original draft. AM: Investigation, Resources, Validation, Writing – review & editing. MiS: Conceptualization, Project administration, Writing – review & editing, Supervision, Validation. PJ: Data curation, Funding acquisition, Validation, Writing – review & editing. ZJ-Z: Validation, Writing – review & editing. MaS: Formal Analysis, Software, Visualization, Writing – review & editing. ZW: Formal Analysis, Software, Visualization, Writing – review & editing. MR-Z: Conceptualization, Project administration, Supervision, Writing – review & editing.
